# Pathologic Implications of Radial Resection Margin and Perineural Invasion to Adjuvant Chemotherapy after Preoperative Chemoradiotherapy and Surgery for Rectal Cancer: A Multi-Institutional and Case-Matched Control Study

**DOI:** 10.3390/cancers14174112

**Published:** 2022-08-25

**Authors:** Soo-Yoon Sung, Sung Hwan Kim, Hong Seok Jang, Jin Ho Song, Songmi Jeong, Ji-Han Jung, Jong Hoon Lee

**Affiliations:** 1 Department of Radiation Oncology, Eunpyeong St. Mary’s Hospital, College of Medicine, The Catholic University of Korea, Seoul 03312, Korea; 2 Department of Radiation Oncology, St. Vincent’s Hospital, College of Medicine, The Catholic University of Korea, Seoul 16247, Korea; 3 Department of Radiation Oncology, Seoul St. Mary’s Hospital, College of Medicine, The Catholic University of Korea, Seoul 06591, Korea; 4 Department of Radiation Oncology, Uijeongbu St. Mary’s Hospital, College of Medicine, The Catholic University of Korea, Seoul 11765, Korea; 5 Department of Hospital Pathology, St. Vincent’s Hospital, College of Medicine, The Catholic University of Korea, Seoul 16247, Korea

**Keywords:** adjuvant chemotherapy, perineural invasion, recurrence, rectal cancer, surgical margin

## Abstract

**Simple Summary:**

The significance of the role of adjuvant chemotherapy in patients with rectal cancer treated with neoadjuvant chemoradiation and surgery has yet to be been determined. In this multi-center and case-matched analysis, we evaluated 1799 patients with rectal cancer who received adjuvant chemotherapy after neoadjuvant chemoradiation and total mesorectal excision. Positive surgical margin and perineural invasion were poor prognostic factors after neoadjuvant chemoradiotherapy and curative surgery. Adjuvant chemotherapy significantly decreased recurrences in patients with positive margin or perineural invasion. The role of adjuvant chemotherapy in rectal cancer patients without positive margin and perineural invasion after neoadjuvant chemoradiotherapy and surgery could be limited.

**Abstract:**

We assessed the exact role of adjuvant chemotherapy after neoadjuvant chemoradiotherapy (CRT) and surgery in rectal cancer patients with positive surgical margin or perineural invasion (PNI). This multi-institutional study included 1799 patients with rectal cancer at cT3-4N0-2M0 stages. Patients were divided into two groups. The high-risk group had a positive margin and/or perineural invasion. The low-risk group showed no positive margin or PNI. Propensity-score matching analysis was performed, and a total of 928 patients, with 464 in each arm, were evaluated. The high-risk group showed significant differences in overall survival (OS, 73.4% vs. 53.9%, *p* < 0.01) and recurrence-free survival (RFS, 52.7% vs. 40.9%, *p* = 0.01) at five years between the adjuvant chemotherapy arm and observation arm. The low-risk group showed no significant differences in 5-year OS (*p* = 0.61) and RFS (*p* = 0.75) between the two arms. Multivariate analyses showed that age, pathologic N stage, and adjuvant chemotherapy were significantly correlated with OS and RFS in the high-risk group (all *p* < 0.05). Adjuvant chemotherapy improved OS and RFS more significantly in rectal cancer patients with positive surgical margin or PNI than in those with negative surgical margin and PNI.

## 1. Introduction

Survival outcomes for rectal cancer have improved greatly over the past decades due to advances in treatment modality and diagnostic tools [1,2,3,4]. According to Surveillance, Epidemiology, and End Results (SEER) Program cancer statistics, the 5-year relative survival rates of patients with rectal cancer increased from 56.3% in 1984–1986 to 69.1% in 2009–2015 [5]. Adoption of total mesorectal excision (TME) and neoadjuvant chemoradiation (CRT) in rectal cancer reduced the local recurrence rates from 30–45% to 5–10% [6,7,8]. However, distant metastasis still occurred in a third of patients with locally advanced rectal cancer who received neoadjuvant CRT and curative TME [9].

The significance of adjuvant chemotherapy after neoadjuvant CRT and TME in patients with rectal cancer has yet to be established, though adjuvant chemotherapy is currently performed in usual clinical settings. Four randomized clinical trials failed to establish the benefits of such treatment definitively [10,11,12]. These findings were reflected in international guidelines, which differed considerably in their recommendations [13,14,15,16]. The patient selection criteria vary even among physicians using adjuvant chemotherapy as a standard treatment, due to the heterogeneity of the patients. The role of adjuvant chemotherapy in different subgroups of patients with rectal cancer is unknown.

Locally advanced rectal cancer patients who had initially clinical T3-4 or N1-2 disease showed heterogenous response to adjuvant chemotherapy. Additional factors besides stage were needed to stratify patients for tailored adjuvant treatment. Decisions regarding adjuvant chemotherapy are typically made after surgical resection. Pathologic features might be the most reflective of disease burden at that time. Thus, we investigated patients who benefited from adjuvant chemotherapy based on pathologic factors. Surgical margin and perineural invasion are well-known prognostic features in rectal cancer [17,18,19,20,21,22,23,24,25,26,27]. We investigated the role of adjuvant chemotherapy in patients with rectal cancer who received neoadjuvant CRT and curative TME in this multi-institutional study, according to the status of surgical margin and perineural invasion.

## 2. Materials and Methods

### 2.1. Patients

Patients with pathologically proven rectal adenocarcinoma at stages T3-4N0-2M0 according to AJCC stage 7th edition were included in this study. Patients with tumors located above 10 cm from anal verge, performance status ≥ ECOG 2, a history of previous malignancy, and patients who did not receive neoadjuvant CRT and curative TME were excluded. Data were collected from 6 institutions between May 2010 and June 2019. The current study was approved by the Institutional Review Board of each participating institute. Informed consent was waived due to the retrospective nature of this study.

### 2.2. Treatments

Initial staging evaluation included digital rectal examination, flexible sigmoidoscopy, rectal MRI imaging, chest and abdominopelvic CT scan, in addition to blood tests, including complete blood count, liver function test, renal function test, and serum carcinoembryogenic antigen (CEA).

Neoadjuvant CRT was performed before surgery. All patients underwent simulation CT scan for radiotherapy. A dose of 45 Gy in 25 fractions was delivered to the whole pelvis, followed by a booster dose of 5.4 Gy in 3 fractions to the gross lesions. Three chemotherapy regimens were used: intravenous 5-fluorouracil (5-FU; 400 mg/m^2^/d) and leucovorin (20 mg/m^2^/d) during the first and fifth weeks, continuous 5-FU (225 mg/m^2^/d), and oral administration of capecitabine (825 mg/m^2^/d) twice daily. Curative TME was performed 4 to 8 weeks after the completion of radiotherapy. Adjuvant chemotherapy was administered 4 to 6 weeks after surgery according to the policy of each participating institution and consisted of (1) four cycles of intravenous 5-FU (425 mg/m^2^/d) and leucovorin (40 mg/m^2^/d) on five consecutive days, (2) six cycles of FOLFOX, a 2-h infusion of oxaliplatin (85 mg/m^2^) and leucovorin (400 mg/m^2^) followed by a bolus injection of 5-FU (400 mg/m^2^); and (3) six cycles of capecitabine (1250 mg/m^2^/d) twice daily for two weeks.

### 2.3. Pathologic Examination

**Figure 1:**
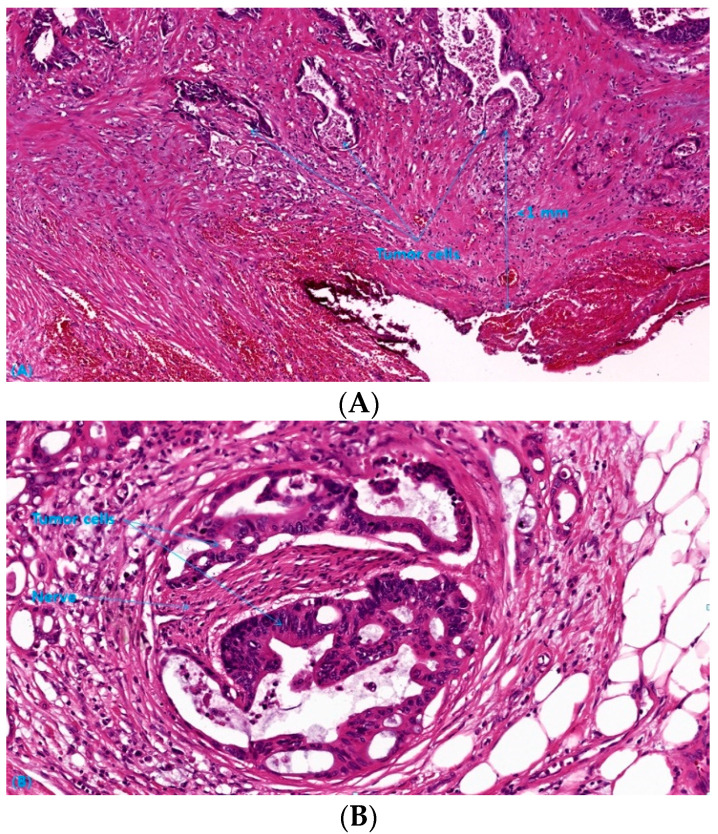
(**A**) Microscopic tumor involvement within 1 mm of the circumferential resection margin (hematoxylin and eosin stain; original magnification ×100) (**B**) Perineural invasion of tumor cells (hematoxylin and eosin stain; original magnification ×200).

Pathologic specimens were assessed using the standardized protocol of the College of American Pathologists by specialized pathologists on colon and rectum in each center [28]. The tumor size, depth of invasion, presence of lymph node metastasis, tumor grade and differentiation, radial resection margin, lymphovascular and perineural invasion, and tumor response to the neoadjuvant chemoradiation were evaluated by the protocol. Positive surgical margin was defined as the presence of tumor cells on ≤1 mm from the circumferential margin of the specimen (Figure 1A) and positive PNI was defined as the presence of tumor cells inside the perineurium (Figure 1B).

### 2.4. Statistical Analyses

Patients were divided into two groups according to the status of surgical margin and PNI based on the pathology report. The high-risk group of patients showed positive surgical margins and/or PNI. The low-risk group had no surgical margins and PNI.

Baseline patient characteristics of high- and low-risk groups were compared using chi-square test for categorical variables and *t*-test for continuous variables. Propensity score matching was performed to balance the difference in patient characteristics between the two groups. PSM was conducted using the nearest neighbor algorithm in a 1:1 manner. Age, sex, pre-CRT CEA, surgery, pathologic T stage, and pathologic N stage were used to calculate propensity scores. The Hosmer–Lemishow test and c-index were used to evaluate the calibration and discrimination of the model.

Overall survival (OS) was defined as the time interval from the date of curative TME surgery to the date of death or last follow-up. Recurrence-free survival (RFS) was defined as the time interval from the date of surgery to the date of recurrence or death. Local recurrence and distant metastasis were defined as recurrences inside and outside the pelvic cavity, respectively. Local recurrence-free survival (LRFS) was defined as the time interval from the date of surgery to the date of local recurrence or death. Distant metastasis-free survival (DMFS) was defined as the time interval from the date of surgery to the date of distant metastasis or death. Survival curves were generated using Kaplan–Meier methods and compared using Log-rank test. Multivariate analyses were performed using Cox proportional-hazards regression. Values of *p* < 0.05 were considered as statistically significant. Statistical analyses were performed using R version 4.0.5 (R Development CoreTeam, Vienna, Austria).

## 3. Results

A total of 1799 patients were evaluated in this study. Positive surgical margins were found in 205 patients (11.4%) and positive PNI occurred in 350 cases (19.5%). Based on the status of surgical margin and PNI, the low-risk group contained 1335 patients (74.2%) and the high-risk group included 464 patients (25.8%). Significant differences were detected based on gender, CEA, surgery, pathologic T stage, pathologic N stage, and histologic grade between the two groups (all *p* < 0.05). To balance the baseline characteristics, 1:1 propensity score matching was performed (Table 1).

Next, 924 patients were allocated to each high-risk group (*n* = 462) or low-risk group (*n* = 462). The matching model was relatively well calibrated. Hosmer–Lemeshow goodness score for this model was 3.640 (*p* = 0.89). The c-index was 0.895 (*p* < 0.01). After matching, the standardized differences were reduced to less than 0.1 in all demographic factors except surgical margin and PNI. No significant differences were observed in age (*p* = 1.00), gender (*p* = 0.94), serum CEA level (*p* = 0.26), surgery type (*p* = 0.38), pathologic T stage (*p* = 1.00), pathologic N stage (*p* = 0.56), or histologic grade (*p* = 0.63). In matched cohort, adjuvant chemotherapy was administered to 423 (91.6%) patients in low-risk group and 416 (90.1%) in high-risk group. The regimen of adjuvant chemotherapy did not significantly differ between the low-risk and high-risk groups; FOLFOX, 8.2% versus 12.1%; LF, 74.1% versus 68.8%; Xeloda, 9.3% versus 9.3%; *p* = 0.17).

**Figure 2:**
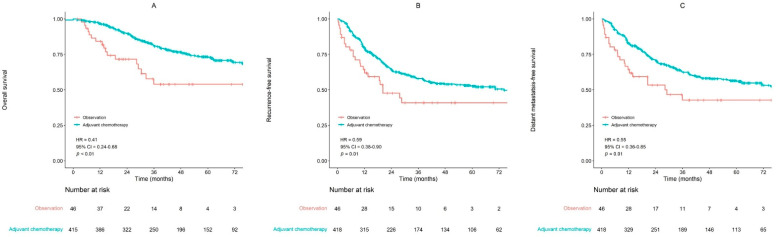
Survival curves in high-risk group. (**A**) Overall survival (**B**) Recurrence-free survival (**C**) Distant metastasis-free survival (HR, hazard ratio; CI, confidence interval).

The 5-year OS and RFS rates in high-risk group at a median follow-up of 47.1 months were 71.4% and 51.4%, respectively. The 5-year OS rates were 73.4% in adjuvant chemotherapy arm and 53.9% in observation arm (Figure 2A). The statistical difference was significant (*p* < 0.01). The 5-year RFS was significantly higher in adjuvant chemotherapy arm than in observation arm (52.7% vs. 40.9%, *p* = 0.01) (Figure 2B). Patients in adjuvant chemotherapy arm also showed significantly improved LRFS (69.2% vs. 49.7%, *p* = 0.01) and DMFS (56.5% vs. 42.8%, *p* = 0.01, Figure 2C) at five years compared with observation arm.

Multivariate analysis revealed that the adjuvant chemotherapy was significantly associated with OS [hazard ratio (HR), 0.39 and 95% of confidence interval (CI), 0.23–0.66; *p* < 0.01] after adjusting for gender, serum CEA, surgery, pathologic T stage, and histologic grade (Table 2).

Other significant prognostic factors were age (*p* < 0.01) and pathologic N stage (*p* < 0.01). Adjuvant chemotherapy (HR, 0.61 and 95% of CI, 0.39–0.94; *p* = 0.03) was also significantly associated with RFS in the multivariate analysis (Table 3).

**Figure 3:**
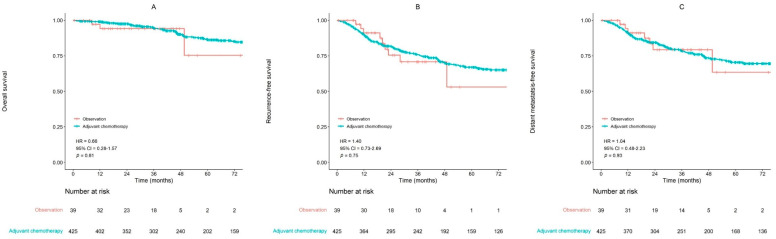
Survival curves in low-risk group. (**A**) Overall survival; (**B**) Recurrence-free survival; (**C**) Distant metastasis-free survival (HR, hazard ratio; CI, confidence interval).

The 5-year OS rates of the low-risk group were 86.6% in adjuvant chemotherapy arm and 75.3% in observation arm (Figure 3A). The difference was not statistically significant (*p* = 0.61). The 5-year RFS rates were not statistically different between the two arms (66.9% vs. 53.1%, *p* = 0.75, Figure 3B). The 5-year LRFS (82.5% vs. 64.4%, *p* = 0.36) and DMFS (70.4% vs. 63.4%, *p* = 0.93, Figure 3C) showed no statistically significant difference.

Age (*p* = 0.04) was a significant prognostic factor for OS in the multivariate analysis. Adjuvant chemotherapy was not significantly associated with OS and RFS in the multivariate analysis (Table 2 and Table 3).

## 4. Discussion

This multi-institutional study revealed that adjuvant chemotherapy improved 5-year OS and RFS significantly in the presence of rectal cancer with positive surgical margin or PNI in patients who received neoadjuvant CRT and TME surgery. Both 5-year LRFS and 5-year DMFS also increased significantly after adjuvant chemotherapy. Patients without positive surgical margin and PNI did not benefit from adjuvant chemotherapy.

The role of adjuvant chemotherapy has been investigated in several randomized trials [9,10,11,12,29,30,31,32]. However, the heterogeneity in the study cohorts and designs makes the interpretation difficult. Most of the trials were conducted without neoadjuvant CRT or TME surgery. Trials performed before and after implementation of neoadjuvant CRT and TME surgery should be reviewed separately. The EORTC 22921 trial involved adjuvant chemotherapy administered to patients who underwent neoadjuvant CRT and TME surgery [9]. Patients were randomized to four treatment groups according to a 2 × 2 factorial design. Neoadjuvant chemotherapy combined with radiotherapy and adjuvant chemotherapy following curative surgery was evaluated. The 5-year OS rate was 67.2% in adjuvant chemotherapy group and 63.2% in surveillance group (*p* = 0.12). Final results after long term follow-up showed a similar outcome. After a median follow-up of 10.4 years, the 10-year OS was 51.8% in adjuvant chemotherapy group and 48.4% in surveillance group (*p* = 0.32). The interpretation of results should consider the poor adherence of patients to adjuvant chemotherapy. Only 42.9% of patients allocated to adjuvant chemotherapy received 95 to 105% of the planned chemotherapy dose without delay, and 26.9% of the patients never started the treatment.

The Quick and Simple and Reliable (QUASAR) trial investigated the benefit of adjuvant chemotherapy in patients with colorectal cancer [10]. Patients with rectal cancer constituted 29% of the entire cohort. Nearly half of patients with rectal cancer underwent neoadjuvant CRT. For rectal cancer patients, the relative risk of recurrence with adjuvant chemotherapy versus observation was 0.68 (95% CI, 0.48–0.96). In QUASAR trial, 77% of patients received at least 80% of their full chemotherapy based on protocol, and only 3% of patients did not start adjuvant chemotherapy. The PROCTOR/SCRIPT trial and the CHRONICLE trial were launched, but ended prematurely due to the slow accrual [11,12]. No difference in OS and DFS was detected between adjuvant chemotherapy group and observation group in both trials.

Given the controversial results of randomized trials, the identification of subgroups benefiting from adjuvant chemotherapy has become an issue. Pathologic features are used as indicators for adjuvant treatment in many other solid organ malignancies. Extracapsular extension of lymph node and positive surgical margins indicate the need for concurrent chemotherapy and adjuvant radiotherapy for head and neck cancer. Presence of metastatic lymph nodes and parametrial invasion represent criteria for adjuvant treatment of cervical cancer. We explored the implications of pathologic features to identify the subgroup of patients with rectal cancer indicated for adjuvant chemotherapy. Surgical margin has been a well-known prognostic factor before neoadjuvant CRT era [3,24,25,26,27]. Quirke et al. first reported that positive surgical margin increased local recurrence in 1986 [25]. A literature review of more than 17,500 patients reported that positive surgical margin is a powerful predictor of local recurrence, distant metastasis and OS after neoadjuvant CRT [24]. The HR of death was 1.7-fold higher in patients with positive surgical margins (95% CI, 1.3 to 2.3). The predictive value of margin was even higher after neoadjuvant CRT compared with no neoadjuvant treatment.

PNI also has been reported as an independent prognostic factor in rectal cancer in several studies [19,20,21,22,23,33]. A meta-analysis showed that HR of PNI was 1.85 for OS in multivariate analysis (95% CI, 1.63–2.12) [20]. In the meta-analysis, 9 studies performed neoadjuvant CRT and 8 studies did not. A 2019 study investigated the prognostic value of PNI in patients who received neoadjuvant CRT [19]. The incidence of PNI was not different between patients treated with and without neoadjuvant CRT (28.3% vs. 29.1%, *p* = 0.79). In patients exposed to neoadjuvant CRT, PNI was an independent prognostic factor for OS in multivariate analysis (HR 2.02, 95% CI 1.10–3.69). Another study reported that, in patients who received neoadjuvant CRT, median DFS was 13.5 months for positive PNI group and 39.8 months for PNI-negative group (*p* < 0.01) [17]. PNI was an independent predictor of DMFS and DFS in that study.

Prognostic value of surgical margin and PNI were evaluated in numerous studies. However, few studies investigated the role of adjuvant chemotherapy according to the pathologic features. This study assessed the value of surgical margin and PNI as indicators for adjuvant chemotherapy in a relatively large size cohort. We demonstrated that patients who completed neoadjuvant CRT and TME surgery might represent a heterogenous population amenable to adjuvant chemotherapy. To establish an optimal strategy for adjuvant chemotherapy, further studies are needed to identify the subgroup that could benefit from adjuvant chemotherapy. These results should be interpreted with caution due to the retrospective nature of the study. To minimize bias, we included patients who received homogenous treatment via neoadjuvant CRT at a dose of 50 Gy and TME. Baseline characteristics were balanced using PSM. Nevertheless, bias regarding patient selection and treatment may exist.

Recently, immunotherapy has been attempted as neoadjuvant and adjuvant therapy for locally advanced rectal cancer. Rectal cancer with deficient mismatch repair (dMMR) is regarded as an immunogenic subtype of colorectal cancer. The dMMR tumors was reported to respond poorly to neoadjuvant CRT [34], but in contrast, showed a good response to immunotherapy. The dMMR rectal cancers have a large mutational burden and an abundance of neoantigens, and the good responsiveness might attribute to those biological features. A prospective phase II trial examined an efficacy of neoadjuvant dostarlimab, an anti-PD-1 monoclonal antibody in patients with stage II-III dMMR rectal cancer [35]. Among 12 patients who completed 6 months of dostarlimab therapy, the clinical complete response was observed in all patients. During the follow-up period of median 12 months, no patients were treated with CRT or surgical resection, because they maintained complete remission states. In another trial, VOLTAGE-A treated microsatellite stable (MSS) rectal cancer patients and dMMR patients with nivolumab, an anti-PD-1 antibody, after neoadjuvant CRT [36]. In preliminary results, MSS rectal cancer patients showed 30% pCR rate (11/37), and dMMR patients showed 60% (3/5).

## 5. Conclusions

The response of patients with positive surgical margin and/or PNI to adjuvant chemotherapy varied compared with those with negative surgical margin and PNI. Adjuvant chemotherapy could more efficiently decrease recurrence and increase survival in patients with pathologic features of radial resection margin and PNI rather than those with negative radial margin and PNI of tumor after neoadjuvant CRT and curative TME.

## Figures and Tables

**Table 1:** Patient and tumor characteristics before and after matching.

	Before Matching				After Matching			
Characteristic-No. (%)	Low-Risk Group (*n* = 1335)	High-Risk Group (*n* = 464)	*p*-Value	SMD	Lowhigh-Risk Group (*n* = 464)	High-Risk Group (*n* = 464)	*p*-Value	SMD
Age			0.21	0.07			1.00	0.01
≤65	823 (61.6%)	302 (65.1%)			301 (64.9%)	302 (65.1%)		
>65	512 (38.4%)	162 (34.9%)			163 (35.1%)	162 (34.9%)		
Gender			0.02	0.13			0.94	0.01
Male	894 (67.0%)	339 (73.1%)			337 (72.6%)	339 (73.1%)		
Female	441 (33.0%)	125 (26.9%)			127 (27.4%)	125 (26.9%)		
CEA, ng/mL			<0.01	0.31			0.26	0.08
≤5	926 (69.4%)	252 (54.3%)			270 (58.2%)	252 (54.3%)		
>5	409 (30.6%)	212 (45.7%)			194 (41.8%)	212 (45.7%)		
Surgery			<0.01	0.19			0.38	0.07
LAR	1229 (92.1%)	400 (86.2%)			410 (88.4%)	400 (86.2%)		
APR	106 (7.9%)	64 (13.8%)			54 (11.6%)	64 (13.8%)		
Pathologic T			<0.01	0.96			1.00	<0.001
pT1-2	715 (53.6%)	60 (12.9%)			60 (12.9%)	60 (12.9%)		
pT3-4	620 (46.4%)	404 (87.1%)			404 (87.1%)	404 (87.1%)		
Pathologic N			<0.01	0.56			0.56	0.04
cN0	1000 (74.9%)	227 (48.9%)			237 (51.1%)	227 (48.9%)		
cN+	335 (25.1%)	237 (51.1%)			227 (48.9%)	237 (51.1%)		
Histologic grade			0.02	0.13			0.63	0.04
Low	1263 (94.6%)	424 (91.4%)			429 (92.5%)	424 (91.4%)		
High	72 (5.4%)	40 (8.6%)			35 (7.5%)	40 (8.6%)		
Surgical margin			<0.01	1.26			<0.01	1.26
Negative	1335 (100.0%)	259 (55.8%)			464 (100.0%)	259 (55.8%)		
Positive	0 (0.0%)	205 (44.2%)			0 (0.0%)	205 (44.2%)		
Perineural invasion			<0.01	2.48			<0.01	2.48
Negative	1335 (100.0%)	114 (24.6%)			464 (100.0%)	114 (24.6%)		
Positive	0 (0.0%)	350 (75.4%)			0 (0.0%)	350 (75.4%)		

APR, abdominoperineal resection; CEA, carcinoembryonic antigen; FU, fluorouracil; LAR, low anterior resection; SMD, standardized mean difference.

**Table 2:** Prognostic factors associated with overall survival in high-risk group and low-risk group.

Variables	High-Risk Group		Low-Risk Group	
	Univariate (*p*) Hazard Ratio (95% CI)	Multivariate (*p*) Hazard Ratio (95% CI)	Univariate (*p*) Hazard Ratio (95% CI)	Multivariate (*p*) Hazard Ratio (95% CI)
Age, year	0.02	<0.01	0.03	0.04
≤65	1		1	1
>65	1.74 (1.22–2.49)	1.87 (1.30–2.69)	1.77 (1.04–2.99)	1.73 (1.02–2.94)
Gender	0.96		0.92	
Male	1		1	
Female	1.01 (0.68–1.49)		0.97 (0.54–1.75)	
CEA, ng/mL	0.82		0.11	
≤5	1		1	
>5	1.04 (0.73–1.48)		1.53 (0.91–2.58)	
Surgery	0.01		0.36	
LAR	1		1	
APR	1.77 (1.16–2.72)		1.39 (0.68–2.84)	
Pathologic T	0.03		0.28	
ypT0-2	1		1	
ypT3-4	2.12 (1.08–4.18)		1.66 (0.66–4.17)	
Pathologic N	<0.01	<0.01	0.04	0.08
ypN0	1	1	1	1
ypN+	2.57 (1.76–3.75)	2.33 (1.57–3.46)	1.76 (1.03–3.00)	1.62 (0.95–2.76)
Histologic grade	0.04		0.17	
Low	1		1	
High	2.05 (1.24–3.38)		1.75 (0.79–3.86)	
Adjuvant Chemotherapy	<0.01	<0.01	0.35	
No	1	1	1	
Yes	0.41 (0.24–0.68)	0.39 (0.23–0.66)	0.66 (0.28–1.57)	

APR abdominoperineal resection, CEA carcinoembryonic antigen, CI confidence interval, LAR low anterior resection.

**Table 3:** Prognostic factors associated with recurrence-free survival in high-risk group and low-risk group.

Variable	High-Risk Group		Low-Risk Group	
	Univariate (*p*) Hazard Ratio (95% CI)	Multivariate (*p*) Hazard Ratio (95% CI)	Univariate (*p*) Hazard Ratio (95% CI)	Multivariate (*p*) Hazard Ratio (95% CI)
Age, year	0.07	0.04	0.51	
≤65	1	1	1	
>65	1.30 (0.98–1.72)	1.34 (1.01–1.77)	1.13 (0.78–1.64)	
Gender	0.14		0.57	
Male	1		1	
Female	1.25 (0.93–1.68)		1.12 (0.76–1.66)	
CEA, ng/mL	0.55		0.06	
≤5	1		1	
>5	1.09 (0.83–1.43)		1.41 (0.99–2.00)	
Surgery	<0.01	<0.01	0.10	
LAR	1	1	1	
APR	1.94 (1.38–2.73)	1.74 (1.21–2.51)	1.50 (0.92–2.45)	
Pathologic T	<0.01		0.01	
ypT0-2	1		1	
ypT3-4	2.11 (1.27–3.52)		2.83 (1.32–6.07)	
Pathologic N	<0.01	<0.01	<0.01	0.02
ypN0	1	1	1	1
ypN1-2	2.04 (1.53–2.70)	1.81 (1.35–2.44)	2.08 (1.44–3.00)	1.53 (1.08–2.16)
Histologic grade	0.04		0.02	
Low	1		1	
High	1.55 (1.01–2.37)		1.87 (1.09–3.21)	
Adjuvant Chemotherapy	0.01	0.03	0.31	
No	1	1	1	
Yes	0.59 (0.38–0.90)	0.61 (0.39–0.94)	1.40 (0.73–2.69)	

APR abdominoperineal resection, CEA carcinoembryonic antigen, CI confidence interval, LAR low anterior resection.

## Data Availability

For original data, please contact the corresponding author.
